# Sensitivity of multi-parametric quantitative magnetic resonance imaging for multiple sclerosis pathology

**DOI:** 10.1371/journal.pone.0318415

**Published:** 2025-04-16

**Authors:** Sarah Schlaeger, Mark Mühlau, Guillaume Gilbert, Irene Vavasour, Thomas Amthor, Mariya Doneva, Aurore Menegaux, Maria Mora, Markus Lauerer, Viola Pongratz, Claus Zimmer, Benedikt Wiestler, Jan S. Kirschke, Christine Preibisch, Ronja C. Berg

**Affiliations:** 1 Department of Diagnostic and Interventional Neuroradiology, School of Medicine and Health, TUM Klinikum Rechts der Isar, Technical University of Munich, Munich, Germany; 2 Department of Radiology, LMU University Hospital, LMU Munich, Munich, Germany; 3 Department of Neurology, School of Medicine and Health, TUM Klinikum Rechts der Isar, Technical University of Munich, Munich, Germany; 4 MR Clinical Science, Philips Healthcare, Mississauga, ON, Canada; 5 Department of Radiology, University of British Columbia, Vancouver, BC, Canada; 6 Philips Research Europe, Hamburg, Germany; University of Rochester, UNITED STATES OF AMERICA

## Abstract

**Background:**

In recent years, quantitative magnetic resonance imaging (MRI) made progress towards clinical applicability mainly through advances in acceleration techniques. In patients with multiple sclerosis (MS), objective quantitative MRI-based characterization of subtle pathological alterations in lesions, perilesion (PL), as well as normal-appearing (NA) white matter (NAWM) and grey matter (NAGM) would revolutionize clinical assessment. While numerous quantitative techniques have been applied in studies of MS patients, their diagnostic significance especially for individual patients with relatively short disease duration is unclear. Therefore, we investigated the sensitivity of several quantitative MRI parameters to focal and diffuse MS pathology in a clinical feasibility study with a small sample size.

**Methods:**

In 13 MS patients with a mean disease duration of 8 years and a mean EDSS of 1.1 as well as 14 healthy age-matched controls (HC), we acquired nine (semi-)quantitative magnetic resonance (MR) biomarkers, namely myelin water fraction (MWF), magnetization transfer (MT) saturation (MTsat), inhomogeneous MT ratio (ihMTR), quantitative longitudinal relaxation time (qT1), intrinsic (qT2) and effective (qT2*) quantitative transverse relaxation times, proton density (PD), quantitative susceptibility mapping (QSM), and the ratio between T1-weighted and T2-weighted images (T1w/T2w). Four volumes of interest were automatically defined (NA/HC grey matter (GM), NA/HC white matter (WM), lesion, and PL), and biomarker values were analyzed between groups and tissue types.

**Results:**

For all nine assessed biomarkers, mean values per patient were significantly different between lesion, PL, and NAWM (*p* <  0.05, FDR corrected). The lesion values of qT1, qT2, qT2 * , PD, and QSM were rather inhomogeneous. Furthermore, MWF, MTsat, and ihMTR were sensitive to diffuse WM pathology in MS with the largest absolute differences between NAWM and HCWM medians, albeit not statistically significant after correction for multiple testing.

**Discussion:**

In our study, we successfully compared nine different quantitative MR parameters within the same subjects for tissue characterization of MS. Our study adds relevant aspects to the current debate on different sensitivities of various quantitative MR biomarkers to MS pathology. While all investigated MR biomarkers allowed characterizing lesions in individual patients, a separation of NAWM and HCWM could be most promising with the myelin-sensitive measures MWF, MTsat, and ihMTR.

## Introduction

To date, magnetic resonance imaging (MRI) is the major tool for the diagnostic work-up of multiple sclerosis (MS) patients [1]. Conventional, weighted MRI sequences allow for the detection of lesions in the central nervous system, which currently represents the main pillar of MS diagnosis [2]. However, the manifold pathological changes in diseased nervous tissue of MS patients go far beyond what can be assessed with routine radiological reading of conventional MRI. In particular, the assessment of subtle changes not only within lesions (histologically ranging from early and late active to chronic active, chronic inactive, and remyelinating lesions [3, 4]), but also within perilesional (PL), normal-appearing grey matter (NAGM), and normal-appearing white matter (NAWM) is hardly possible based on qualitative magnetic resonance (MR) contrasts [5, 6]. While NAGM and NAWM tissue does not show obvious damage on qualitative MRI, it has been shown to be affected by complex microstructural alterations due to diffuse microglia activation and Wallerian degeneration [7–9]. In the immediate area around lesions, the PL region, significant pathological changes such as myelin and axonal damage are present, less obvious than in lesions, but considerably different from more distant NAGM or NAWM [10, 11].

In research settings, quantitative MRI methods have been widely used to investigate MS allowing for the determination of microstructural alterations with high sensitivity and both low inter-observer and inter-scanner variability [6]. Ideally, quantitative techniques are comparable between acquisition sites and longitudinal examinations, and promise to provide objective, absolute tissue parameters allowing for the detection of even subtle tissue alterations. Thus, they enable a more profound characterization of pathological changes in lesions, PL, NAGM, and NAWM [12].

Over the years, a wide range of different quantitative MRI contrasts has been explored, revealing numerous, often complementary, insights into the complex pathological changes in MS brain tissue [6]. Given the demyelinating nature of MS, myelin-sensitive techniques appear particularly promising. Among these, myelin water imaging, which determines the myelin water fraction (MWF) by measuring the quickly decaying signal arising from water trapped between myelin sheaths, is most established [13]. Also, measurements of the characteristic signal decrease due to the magnetization transfer (MT) effect between protons in macromolecules (such as myelin) and free water [14] have been found to correlate with the myelin concentration [15–17]. In this regard, two promising MT variants are based on either a single saturation pulse (MTsat) or exploit the dipolar order relaxation time associated with myelinated structures (ihMTR). Additionally, information on the myelin content can be derived from the macromolecular tissue volume, a measure of the macromolecule concentration that can be calculated from measurements of the proton density (PD) [18]. A totally different approach is offered by quantitative susceptibility mapping (QSM), which allows determining the spatial distribution of tissues with different magnetic susceptibilities from distortions of the magnetic field. It is affected by both myelin integrity and iron content [5,19]. Beyond that, quantification of the longitudinal relaxation time T1 (qT1), as well as intrinsic T2 (qT2) and effective T2 * (qT2*) transverse relaxation times is of particular interest, as these parameters are sensitive to a variety of pathological changes associated with myelin, axons, iron, and water content [20]. Finally, at least in white matter tissue, the semi-quantitative ratio between T1-weighted and T2-weighted images (T1w/T2w) has been found to be related to myelin content [21].

Recently, advances with respect to imaging acceleration facilitate the determination of quantitative MR biomarkers within clinically feasible time frames, and the translation of quantitative MRI from research to clinical application is imminent. However, it is still unknown which of the quantitative MR measures are the most sensitive to particular structural alterations and may be applicable for diagnosis and follow up in individual patients. Only a few studies have evaluated how these quantitative measures compare to one another regarding their ability to detect and describe MS pathology in individual patients [22–26]. In particular, comparative, multi-parametric datasets from patients with short disease courses and low disease burden are rare. However, this patient cohort is of major interest because it represents the main target group for disease-modifying drugs with lifelong longitudinal MRI examinations.

Therefore, in a clinical feasibility study with a small sample size, we assessed nine different (semi-)quantitative MR biomarkers in MS patients with low Expanded Disability Status Scale scores (EDSS) (mean 1.1) compared to healthy controls (HC), investigating which MR biomarkers allow for the most sensitive characterization of different pathological hallmarks in MS brain tissue. The aim was to identify which MR biomarkers could be used for the evaluation of individual patients and thus are most promising for an implementation in the clinical routine. Given our focus on finding an MR biomarker with sufficient diagnostic significance for the individual MS patient, we decided to perform a comprehensive study on a small cohort, where the diagnostic potential of a large number of different quantitative MR biomarkers was assessed by an extensive scanning procedure of each participant. On a subject- and group-level, we analyzed the MR biomarkers for their ability to discriminate both between NAGM and HC grey matter (HCGM) as well as between NAWM and HC white matter (HCWM). We compared lesion, PL, and NAWM tissue of MS patients. Furthermore, for each MR biomarker, we investigated – on a lesion-level – the variance of lesion parameter values compared to surrounding tissue.

## Methods

### Study population

The study population comprises 13 patients with MS and 14 age-matched HC who were recruited between July 01, 2020 and February 28, 2022. Demographic and clinical details are provided in Table 1. Twelve of the 13 MS patients were treated with disease-modifying drugs. All participants provided written informed consent for participation prior to the MRI examination. The study design was approved by the local ethics commission of the Klinikum rechts der Isar, Technical University of Munich (TUM). In line with the local ethics guidelines and participant privacy policies, data cannot be shared publicly. Acquired data will be shared upon reasonable request and based on a formal data sharing agreement.

**Table 1:** Demographic and clinical details of study population.

	MS patients (n = 13)	Healthy controls (n = 14)
Gender	8 f/ 5 m	11 f/ 3 m
Age [years]	34.3 ± 8.2 (23; 46)	31.9 ± 7.9 (23; 47)
MS subtype	RRMS n = 11; SPMS n = 1; CIS n = 1	–
EDSS	1.1 ± 1.3 (0; 4.5)	–
Disease duration [years]	8 ± 5 (1; 15)	–
Lesion volume [mm³]	34.6 ± 15.75 (75; 1007)	–

Age, EDSS, disease duration, and lesion volume are provided as mean ±  standard deviation followed by the range in brackets. CIS, clinically isolated syndrome; EDSS, Expanded Disability Status Scale; MS, multiple sclerosis; RRMS, relapsing remitting MS; SPMS, secondary progressive MS.

### MRI measurements and parameter map calculations

Each participant’s brain was scanned on a 3 T MRI system (Ingenia Elition X, Philips Healthcare, Best, The Netherlands; release 5.6.1) using a 32-channel head coil. The scan protocol included a T1w magnetization prepared rapid gradient echo (MPRAGE), a T2w fluid-attenuated inversion recovery (FLAIR), and the following sequences for quantitative MR biomarker assessment: 3D gradient and spin echo (GRASE) with 48 echoes for mapping of the myelin water fraction (MWF) [27] and qT2, 3D gradient echo with three echoes and the MT-pulses for calculating the inhomogeneous MT ratio (ihMTR) [28], three 3D multi-echo gradient echo data sets with T1-, proton density (PD)-, and MT-weighting, including B1-mapping, to calculate MTsat, qT1, qT2 * , and PD using the hMRI toolbox [29], and a 3D gradient echo sequence with six echoes for QSM using the MEDI toolbox [30]. T1w/T2w was calculated based on the MPRAGE data and the 15th echo (TE =  120 ms) of the GRASE data.

**Fig 1:**
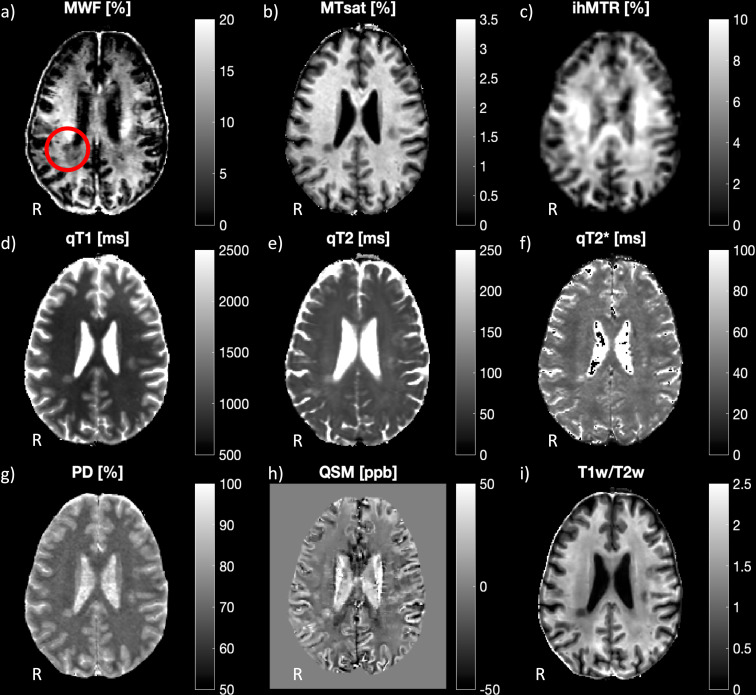
Representative parameter maps for all nine MR biomarkers in an MS patient. a) myelin water fraction (MWF), b) magnetization transfer saturation (MTsat), c) inhomogeneous MT ratio (ihMTR), d) qT1, e) qT2, f) qT2 * , g) proton density (PD), h) quantitative susceptibility mapping (QSM), and i) ratio between T1-weighted and T2-weighted images (T1w/T2w). A representative periventricular lesion is indicated by a red circle in the MWF map. Orientation is indicated with R for right.

In each participant, MPRAGE and FLAIR sequences were scanned first. The order of all other scans was permuted across participants. Scan parameters are provided in S1 Table. In one HC participant, no QSM scan could be performed due to patient discomfort at the end of the scanning procedure. Parameter map calculations for all nine assessed biomarkers were performed using Matlab (R2020a, Mathworks, Natick, MA, USA). Please refer to the supplementary material for an overview of the postprocessing routines (S1 Appendix). Representative quantitative parameter maps for all nine MR biomarkers are shown in Fig 1.

### Volume of interest definition

**Fig 2:**
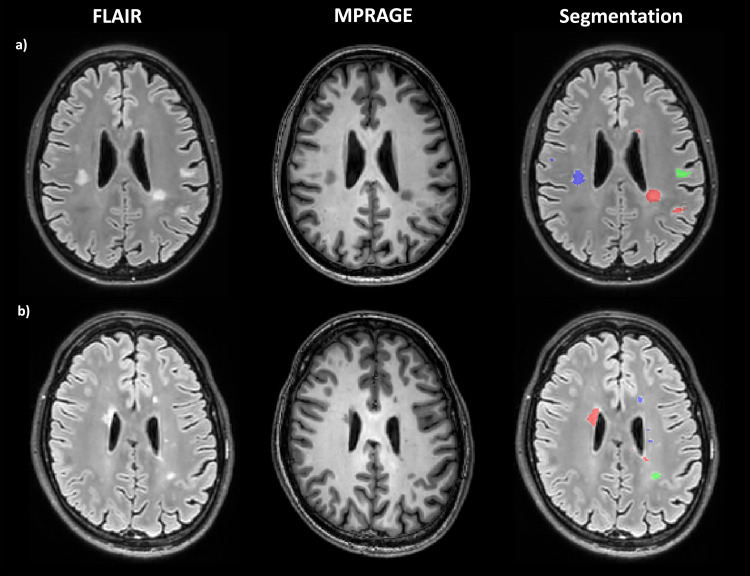
Representative lesion segmentation of two MS patients (a, b). **FLAIR and MPRAGE images are provided for comparison.** Orientation is indicated with R for right. Colors indicate location (red: periventricular; green: (juxta)cortical; blue: subcortical). FLAIR, fluid-attenuated inversion recovery; MPRAGE, T1w magnetization prepared rapid gradient echo.

Lesions, defined as white matter hyperintensities on FLAIR images, were automatically segmented using an in-house built U-net [31] (Fig 2). The segmentation was performed on the individuals’ MPRAGE data and verified by one neuroradiologist (six years of experience). Every lesion segmentation mask was eroded by one voxel, resulting in n =  322 lesion segmentations from 13 MS patients. The resolution of the MPRAGE and thus the lesion and PL volumes is 1x1x1 mm³. All other parameter maps were co-registered to the individual subjects’ MPRAGE data sets. After co-registration of the parameter maps to the MPRAGE data, the lesion masks (which were created in MPRAGE space) were applied to the quantitative parameter maps.

Whole-brain grey matter (GM) and white matter (WM) masks were derived from lesion-filled MPRAGE data using the “segment” module of SPM12 (version v7771; www.fil.ion.ucl.ac.uk/spm/software/spm12/) thresholded at respective tissue probabilities >  0.9.

Voxels within lesion segmentation masks were excluded from all non-lesion volumes of interest (VOIs).

A two-voxel-wide shell of NAWM surrounding each lesion was defined as PL tissue.

Additionally, three one-voxel-wide shells were defined around the PL VOIs, referred to as shell 1-3. For each shell, a sphere-shaped structuring element of radius n voxel(s) was added to the PL mask. Lesion mask, PL mask, and shell mask n-1 were excluded and the resulting shell mask n was intersected with the whole-brain WM segmentation. A detailed description of the VOI definitions is provided in the supplementary material (S2 Appendix).

The evaluation of MR biomarker values in all described VOIs was performed within a subject-specific common imaging volume that was covered by all applied MRI measurements. Thereby, most of the defined VOIs slightly deceased in size and some of the segmented lesions (mostly in the infratentorial part of the brain) were excluded from the analysis.

### Quantitative analysis

Parameter maps were co-registered to the individual subjects’ MPRAGE data using trilinear interpolation and analyses were performed on a subject-, group-, and lesion-level.

For the subject-level analysis, two VOIs were analyzed in each HC participant (HCWM and HCGM) and four VOIs in each MS participant (lesion, PL, NAGM, and NAWM). In each VOI, mean values of all (semi-)quantitative MR biomarkers were extracted for each participant. Thereby, on a subject-level, an average lesion and PL value was calculated across all lesion segmentations per MS participant (referred to as avgLesion and avgPL). MR biomarker values were compared using a boxplot analysis, in which each data point represented the average MR biomarker value per participant in the respective VOI (HCWM, NAWM, HCGM, NAGM, avgLesion, and avgPL), resulting in 13 data points for MS patients and 14 data points for HC per boxplot. To visualize differences in quantitative values between avgLesion, avgPL, NAWM, and NAGM on an individual-patient basis, line plots were created indicating the average signal of the respective VOI in each individual MS patient.

On a group-level, cohort median values based on the MR biomarker mean values per study participant (13 for MS patients and 14 for HC) were analyzed in avgLesion, avgPL, and NA/HCWM.

For the lesion-level analysis, the MR biomarkers were evaluated in five different VOIs: lesion, PL, and shell 1-3. Thereby, each lesion segmentation (and corresponding PL, and shell VOIs) was treated separately and average parameter values were extracted for each individual VOI (lesion, PL, or shell). These data were represented in violin plots and in line plots to assess

the variance of lesion parameter values compared to surrounding tissue.

In the following, the terms subject-, group-, and lesion-level are summarized to enhance clarity:

- Subject-level: Biomarker values for different anatomical regions (GM, WM, lesion, PL) were analyzed separately for each individual. This resulted in 13 data points for MS patients and 14 data points for HC for each biomarker and anatomical region. Each subject contributed a single mean value for each anatomical region.
- Group-level: The median of the mean values across all subjects was calculated for each anatomical region, separately for MS patients and HC. This approach yielded one data point per biomarker and anatomical region for each group.
- Lesion-level: Each segmented lesion was analyzed individually, resulting in 322 data points. In this analysis, mean biomarker values were calculated from the voxel values within the lesion, PL, and shells 1–3 surrounding the lesion. Homogeneity of lesion values was assessed by investigating the variance of lesion parameter values compared to surrounding tissue.

### Statistical analysis

Statistical analyses were performed with SPSS (version 27.0, IBM SPSS Statistics for MacOS, IBM Corp., Armonk, NY, USA) and Microsoft Excel (2021). A *p*-value of 0.05 was set as threshold for statistical significance. Correction for multiple testing was performed using the Benjamini Hochberg procedure for controlling the false discovery rate (FDR).

A Mann-Whitney U test for nonparametric, independent samples was used to assess significance of difference between MR biomarker values in HCWM, NAWM, HCGM, and NAGM. A Wilcoxon signed-rank test for nonparametric, dependent samples was used to assess the significance of difference between MR biomarker values in avgLesion, avgPL, NAWM, and NAGM.

## Results

### Subject- and group-level analysis

#### Diffuse MS pathology: Characterization of whole-brain WM and GM.

**Fig 3:**
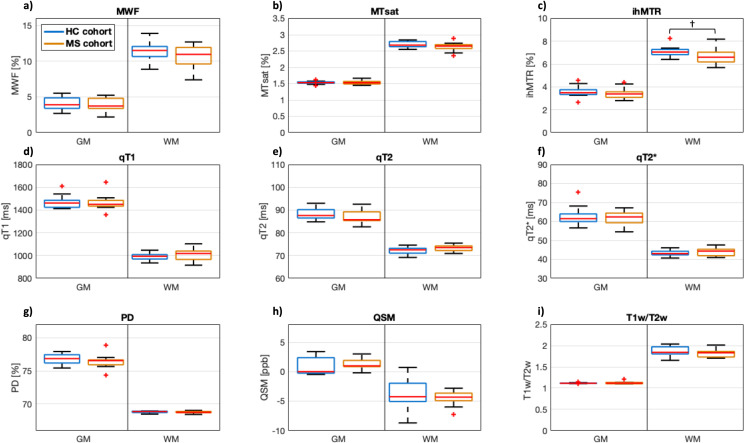
Subject-level analysis of all nine MR biomarkers in grey and white matter (GM/WM) of healthy control (HC) (blue) and multiple sclerosis (MS) patients (orange) using boxplots. Each boxplot represents the mean values from all study participants in the respective volume of interest: a) myelin water fraction (MWF), b) magnetization transfer saturation (MTsat), c) inhomogeneous MT ratio (ihMTR), d) qT1, e) qT2, f) qT2 * , g) proton density (PD), h) quantitative susceptibility mapping (QSM), and i) ratio between T1-weighted and T2-weighted images (T1w/T2w). † indicates statistically significant differences between HC and MS at ****p**** <  0.05. Note that only ihMTR was significantly lower in normal-appearing WM than in HCWM (*p* <  0.05), but did not survive correction for multiple testing.

The myelin-sensitive MR biomarkers MWF, MTsat, and ihMTR were on average slightly lower in white matter of MS patients compared to HCs, while qT1, qT2, and qT2 * were slightly higher (Fig 3, Table 2). The difference in ihMTR values between NAWM and HCWM was significant (*p* =  0.048) but did not survive correction for multiple testing (q-value: 0.006). PD, QSM, and T1w/T2w did not allow for a differentiation between NAWM and HCWM based on a visual assessment of the boxplots (Fig 3) and the corresponding median MR biomarker values (Table 2). Overall, all nine MR biomarkers showed a significant overlap in the subjects’ mean parameter values between HCWM and NAWM (Fig 3), which prevents identification of MS patients based on their MR biomarker values in NAWM tissue. Furthermore, none of the MR biomarkers were significantly different between NAGM and HCGM (Fig 3, Table 2).

**Table 2:** Parameter values in healthy and NAGM and NAWM.

	Grey matter			White matter		
	**HC** **median (range)**	**MS („normal-appearing”)** **median (range)**	**p-value**	**HC** **median (range)**	**MS („normal-appearing”)** **median (range)**	**p-value**
**MWF [%]**	**3.89** (2.68; 5.51)	**3.72** (2.18; 5.23)	0.756	**11.50** (8.87; 13.91)	**10.97** (7.40; 12.70)	0.325
**MTsat [%]**	**1.53** (1.44; 1.63)	**1.53** (1.45; 1.67)	0.943	**2.68** (2.55; 2.84)	**2.65** (2.36; 2.88)	0.259
**ihMTR [%]**	**3.49** (2.64; 4.59)	**3.38** (2.80; 4.42)	0.220	**7.05** (6.40; 8.26)	**6.60** (5.69; 8.18)	**0.048** ^†^
**qT1 [ms]**	**1461.40** (1412.30; 1607.20)	**1450.80** (1358.10; 1645.50)	0.867	**993.50** (935.60; 1047.00)	**1018.30** (915.30; 1103.30)	0.325
**qT2 [ms]**	**87.60** (84.81; 92.96)	**85.74** (82.63; 92.55)	0.325	**72.51** (69.17; 74.57)	**73.53** (70.92; 75.45)	0.068
qT2^*^ **[ms]**	**61.43** (56.56; 75.27)	**62.38** (54.52; 67.13)	0.650	**43.01** (40.72; 46.12)	**44.34** (40.94; 47.59)	0.202
**PD [%]**	**76.85** (75.43; 77.92)	**76.55** (74.33; 78.91)	0.350	**68.84** (68.47; 68.95)	**68.73** (68.41; 69.02)	0.402
**QSM [ppb]**	**0.00** (-0.48; 3.40)	**0.98** (-0.21; 3.00)	0.479	**-4.28** (-8.73; 0.72)	**-4.33** (-7.28; -2.82)	0.579
**T1w/T2w**	**1.11** (1.09; 1.15)	**1.12** (1.10; 1.21)	0.793	**1.84** (1.65; 2.04)	**1.83** (1.71; 2.02)	0.519

Group-level analysis. Median (range) values of all nine MR biomarker mean values across either HC study participants (HCGM and HCWM) or MS patients (NAGM and NAWM).

† indicates statistical significance between HC und MS at *p* <  0.05. HC, healthy control; ihMTR, inhomogeneous MT ratio; MS, multiple sclerosis; MTsat, magnetization transfer saturation; MWF, myelin water fraction; PD, proton density; QSM, quantitative susceptibility mapping; T1w/T2w, ratio between T1-weighted and T2-weighted images.

#### Focal MS pathology: Identification of lesion and PL.

**Fig 4:**
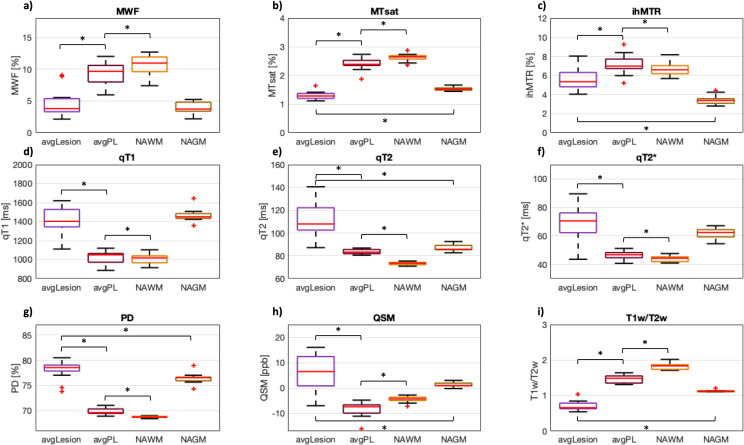
Subject-level analysis of all nine MR biomarkers in lesion (“avgLesion”, purple), perilesion (PL; “avgPL”, brown), normal-appearing white matter (NAWM, orange), and normal-appearing grey matter (NAGM, ochre) of multiple sclerosis patients using boxplots. Each boxplot represents the mean values per study participant in the respective volume of interest: a) myelin water fraction (MWF), b) magnetization transfer saturation (MTsat), c) inhomogeneous MT ratio (ihMTR), d) qT1, e) qT2, f) qT2 * , g) proton density (PD), h) quantitative susceptibility mapping (QSM), and i) ratio between T1-weighted and T2-weighted images (T1w/T2w). For all nine biomarkers, mean values per patient were significantly different between avgLesion, avgPL, and NAWM (*p* <  0.05, FDR corrected). For MTsat, ihMTR, qT2, PD, QSM, and T1w/T2w mean values per patient were also significantly different between avgLesion and NAGM (*p* <  0.05, FDR corrected). *  indicates statistical significance at ****p**** <  0.05, FDR corrected.

**Fig 5:**
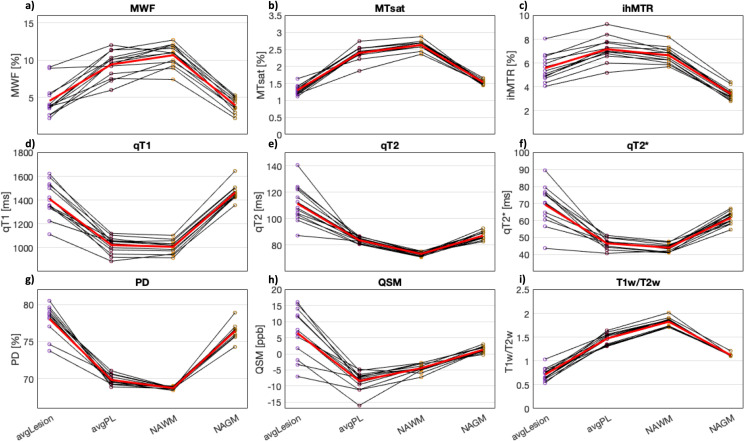
Subject-level analysis showing all nine MR biomarkers in different tissue types of multiple sclerosis patients using line plots. Line plots indicate the mean signal of avgLesion, avgPL, normal-appearing white matter (NAWM), and normal-appearing grey matter (NAGM) in each individual multiple sclerosis (MS) patient (n =  13). The red line indicates the mean signal across all 13 patients. a) myelin water fraction (MWF), b) magnetization transfer saturation (MTsat), c) inhomogeneous MT ratio (ihMTR), d) qT1, e) qT2, f) qT2 * , g) proton density (PD), h) quantitative susceptibility mapping (QSM), and i) ratio between T1-weighted and T2-weighted images (T1w/T2w). In each individual MS patient, all biomarkers showed a clear relation between avgLesion and avgPL values: either all avgPL values were higher than the avgLesion values (for MWF, MTsat, ihMTR, and T1w/T2w) or all avgPL values were lower than the avgLesion values (for qT1, qT2, qT2 * , PD, and QSM).

For all nine biomarkers, mean values per patient were significantly different between avgLesion, avgPL, and NAWM (*p* <  0.05, FDR corrected). For MTsat, ihMTR, qT2, PD, QSM, and T1w/T2w, mean values per patient were also significantly different between lesions and NAGM (*p* <  0.05, FDR corrected) (Table 3). Values of avgPL were always between avgLesion and NAWM values, except for ihMTR (avgPL highest value) and QSM (avgPL lowest value) (Fig 4). MTsat, PD, qT2, and T1w/T2w allowed for a clear separation between lesion and PL, as all PL values lay outside the range of values within lesions (Table 3). In each individual MS patient, all MR biomarkers showed a clear relation between avgLesion and avgPL values: either all avgPL values were higher than the avgLesion values (for MWF, MTsat, ihMTR, and T1w/T2w) or all avgPL values were lower than the avgLesion values (for qT1, qT2, qT2 * , PD, and QSM) (Fig 5).

**Table 3:** Parameter values in different tissue types of MS patients.

	Lesion median (range)	PL median (range)	NAWM median (range)	NAGM median (range)	p-value lesion vs. PL	p-value lesion vs. NAWM	p-value lesion vs. NAGM	p-value PL vs. NAWM	p-value PL vs. NAGM
**MWF [%]**	**3.79** (2.14; 9.06)	**9.67** (5.95; 12.02)	**10.97** (7.40; 12.70)	**3.72** (2.18; 5.23)	**0.001** ^*^	**0.001** ^*^	0.422	**0.007** ^*^	**0.001** ^*^
**MTsat [%]**	**1.28** (1.11; 1.64)	**2.39** (1.87; 2.74)	**2.65** (2.36; 2.88)	**1.53** (1.45; 1.67)	**0.001** ^*^	**0.001** ^*^	**0.003** ^*^	**0.001** ^*^	**0.001** ^*^
**ihMTR [%]**	**5.35** (4.05; 8.05)	**7.00** (5.20; 9.25)	**6.60** (5.69; 8.18)	**3.38** (2.80; 4.42)	**0.001** ^*^	**0.001** ^*^	**0.001** ^*^	**0.007** ^*^	**0.001** ^*^
**qT1 [ms]**	**1403.50** (1111.50; 1620.10)	**1050.30** (886.60; 1120.00)	**1018.30** (915.30; 1103.30)	**1450.80** (1358.10; 1645.50)	**0.001** ^*^	**0.001** ^*^	0.196	**0.039** ^*^	**0.001** ^*^
**qT2 [ms]**	**107.92** (87.20; 140.60)	**83.24** (80.55; 86.84)	**73.53** (70.92; 75.45)	**85.74** (82.63; 92.55)	**0.001** ^*^	**0.001** ^*^	**0.001** ^*^	**0.001** ^*^	**0.016** ^*^
qT2^*^ **[ms]**	**70.51** (43.58; 89.54)	**46.74** (40.68; 51.25)	**44.34** (40.94; 47.59)	**62.38** (54.52; 67.13)	**0.001** ^*^	**0.001** ^*^	0.055	**0.002** ^*^	**0.001** ^*^
**PD [%]**	**78.54** (73.76; 80.50)	**69.61** (68.91; 71.06)	**68.73** (68.41; 69.02)	**76.55** (74.33; 78.91	**0.001** ^*^	**0.001** ^*^	**0.011** ^*^	**0.001** ^*^	**0.001** ^*^
**QSM [ppb]**	**6.51** (-7.02; 16.09)	**-7.45** (-16.08; -4.80)	**-4.33** (-7.28; -2.82)	**0.98** (-0.21; 3.00)	**0.001** ^*^	**0.002** ^*^	**0.028** ^*^	**0.002** ^*^	**0.001** ^*^
**T1w/T2w**	**0.65** (0.54; 1.04)	**1.48** (1.30; 1.64)	**1.83** (1.71; 2.02)	**1.12** (1.10; 1.21)	**0.001** ^*^	**0.001** ^*^	**0.001** ^*^	**0.001** ^*^	**0.001** ^*^

Group-level analysis. Median (range) values of all nine MR biomarker mean values across MS patients in lesion, PL, NAWM, and NAGM.

*  indicates statistical significance at *p* <  0.05, FDR corrected. ihMTR, inhomogeneous MT ratio; MS, multiple sclerosis; MTsat, magnetization transfer saturation; MWF, myelin water fraction; NAGM, normal-appearing grey matter; NAWM, normal-appearing white matter; PL, perilesion; PD, proton density; QSM, quantitative susceptibility mapping; T1w/T2w, ratio between T1-weighted and T2-weighted images.

### Lesion-level analysis

**Fig 6:**
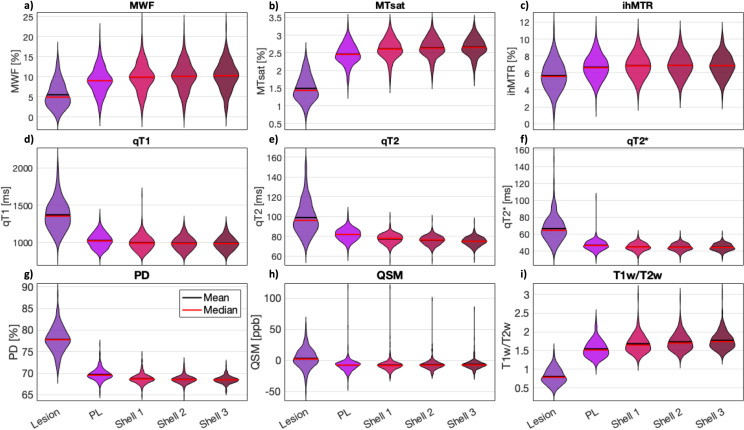
Lesion-level analysis using violin plots with mean (black) and median (red) values to illustrate biomarker inhomogeneity within individual lesions compared to their surrounding tissue including perilesion (PL) and shell 1-3. a) myelin water fraction (MWF), b) magnetization transfer saturation (MTsat), c) inhomogeneous MT ratio (ihMTR), d) qT1, e) qT2, f) qT2 * , g) proton density (PD), h) quantitative susceptibility mapping (QSM), and i) ratio between T1-weighted and T2-weighted images (T1w/T2w). Particularly, the lesion biomarker values for qT1, qT2, qT2 * , PD, and QSM were more heterogeneous than the corresponding biomarker values within the surrounding tissue.

Violin plots were generated for lesion, PL, and shell 1-3 to illustrate biomarker inhomogeneity within individual lesions compared to their surrounding tissue (PL, shell 1-3) (Fig 6). In general, the results of the lesion-level analysis confirmed the findings from subject- and group-level analyses. They showed that the trends in biomarker value decrease or increase continue beyond the PL towards the periphery. All violin plots appeared slender and elongated for MWF and ihMTR, indicating a variance of biomarker values between different lesions as well as for each type of VOI surrounding lesions, between the surrounding tissue of each lesion. All violin plots for MTsat and T1w/T2w appeared less slender and less elongated, yet lesion biomarker values showed comparable variance to the values of surrounding tissue. In contrast, the violin plots for qT1, qT2, qT2 * , and PD lesion values appeared more slender and more elongated than the corresponding violin plots for the surrounding tissue, which tended to be small and bulbous. This indicates that biomarker values of different lesions were more heterogeneous than the biomarker values in the surrounding tissue. The same was true for QSM but with more outlier values for the surrounding tissue. Corresponding mean and standard deviation values are shown in Table 4. For a more detailed illustration of the spatial signal behavior from lesion to surrounding tissue for each individual lesion, supplementary S1 Fig shows the biomarker signal behavior from individual lesions to surrounding tissue plotted as lines with the y-axes scaled to the mean ±  twice the standard deviation of each MR biomarker.

**Table 4:** Parameter values within and surrounding lesions.

	Lesion mean (std)	PL mean (std)	Shell 1 mean (std)	Shell 2 mean (std)	Shell 3 mean (std)
**MWF [%]**	**5.52** (3.17)	**9.03** (3.28)	**9.87** (3.51)	**10.11** (3.53)	**10.23** (3.53)
**MTsat [%]**	**1.49** (0.36)	**2.47** (0.29)	**2.61** (0.29)	**2.65** (0.28)	**2.67** (0.26)
**ihMTR [%]**	**5.69** (1.72)	**6.69** (1.40)	**6.87** (1.38)	**6.89** (1.35)	**6.85** (1.33)
**qT1 [ms]**	**1371.75** (201.32)	**1027.18** (97.02)	**997.21** (100.07)	**990.54** (90.55)	**989.05** (88.54)
**qT2 [ms]**	**99.05** (16.66)	**81.88** (6.14)	**77.52** (5.04)	**76.05** (4.65)	**75.09** (4.43)
qT2^*^ **[ms]**	**66.44** (15.04)	**46.65** (5.91)	**44.81** (4.65)	**44.49** (4.33)	**44.36** (4.08)
**PD [%]**	**77.84** (2.95)	**69.70** (1.37)	**68.75** (1.15)	**68.58** (0.99)	**68.50** (0.89)
**QSM [ppb]**	**3.23** (15.85)	**-7.70** (10.91)	**-7.60** (9.99)	**-6.96** (8.92)	**-6.50** (8.18)
**T1w/T2w**	**0.80** (0.21)	**1.54** (0.22)	**1.68** (0.24)	**1.73** (0.24)	**1.77** (0.24)

Mean and standard deviation of all nine MR biomarker mean values of all individual lesions, PL, and shell 1 to 3 around PL VOIs. ihMTR, inhomogeneous MT ratio; MTsat, magnetization transfer saturation; MWF, myelin water fraction; PL, perilesion; PD, proton density; QSM, quantitative susceptibility mapping; std, standard deviation; T1w/T2w, ratio between T1-weighted and T2-weighted images.

### Diagnostic potential of quantitative MR biomarkers for characterizing MS pathology

**Fig 7:**
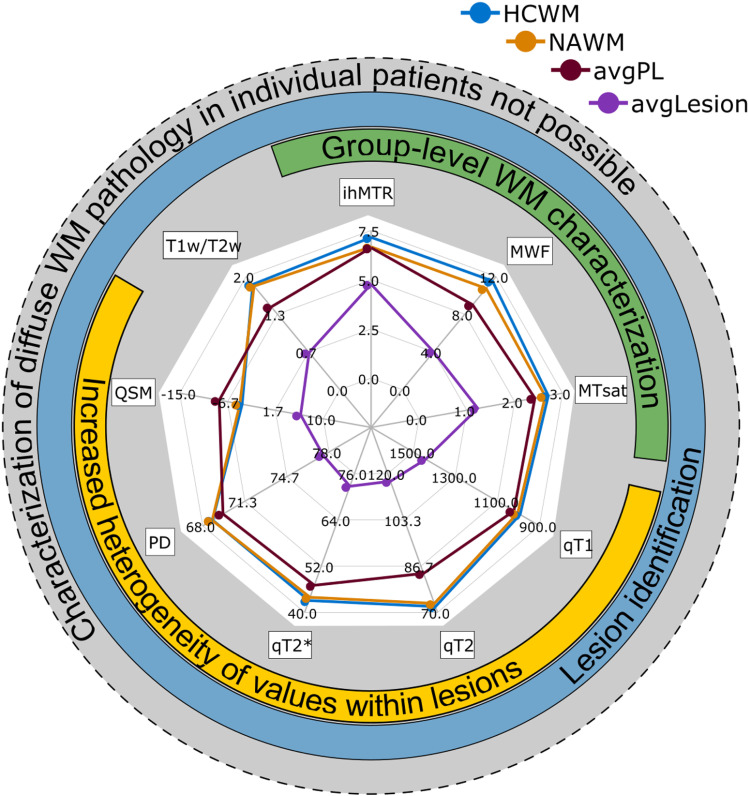
Spider web plot showing the cohort-median values based on the MR biomarker mean values per study participant in healthy control white matter (HCWM, blue), normal-appearing white matter (NAWM, orange), avgPL (red), and avgLesion (purple). Based on the results of this study, the myelin-sensitive markers (MWF, MTsat, and ihMTR) were found to show the largest absolute differences between group medians for characterization of NAWM compared to HCWM on a group-level (green ring). All nine MR biomarkers allowed for lesion detection with significantly different values between avgLesion, avgPL, and NAWM on a group-level (blue ring). For a visual overview of the study’s results the additional yellow ring marks MR biomarkers with increased heterogeneity of values within lesions on a lesion-level (namely qT1, qT2, qT2 * , PD, and QSM). The outermost ring underlines that on a subject-level characterization of diffuse WM pathology in individual patients was not possible. ihMTR, inhomogeneous MT ratio; MWF, myelin water fraction; MTsat, magnetization transfer saturation; PD, proton density; QSM, quantitative susceptibility mapping; T1w/T2w, ratio between T1-weighted and T2-weighted images.

The spider web plot in Fig 7 summarizes the above-described results and provides an overview of the diagnostic potential of the assessed MR biomarkers for characterizing MS pathology. Cohort-median values based on the MR biomarker mean values per study participant in HCWM, NAWM, avgPL, and avgLesion are shown (please also refer to Tables 2 and 3). All nine MR biomarkers allowed for lesion detection with significantly different values between avgLesion, avgPL, and NAWM on a group-level (blue ring). The myelin-sensitive markers (MWF, MTsat, and ihMTR) were best suited for characterization of NAWM compared to HCWM on a group-level (green ring), with the largest absolute differences between group medians, albeit not statistically significant after correction for multiple testing.

## Discussion

In the present quantitative multi-parametric MRI clinical feasibility study, we successfully compared nine different quantitative MR techniques within the same subjects for tissue characterization of MS. By investigating an extensive range of different MR biomarkers in a small study cohort, we add relevant aspects to the current debate on different sensitivities of various quantitative MR biomarkers to MS pathology. Such analyses are necessary to identify which MR biomarkers are most promising for an implementation in clinical routines.

While all investigated MR biomarkers allowed characterizing lesions in individual patients, a separation of NAWM and HCWM could be most promising with the myelin-sensitive measures MWF, MTsat, and ihMTR. A visual overview of the performed study and its results is depicted in S2 Fig.

In more detail, we focused on the biomarkers’ potential to assess the two major, MR-detectable, pathological changes in MS brain tissue: i) focal MS pathology (lesions and PL), and ii) diffuse MS pathology within whole-brain NAWM and NAGM. We could show that the MR biomarkers MWF, MTsat, ihMTR, qT1, qT2, qT2 * , PD, QSM, and T1w/T2w allow for a significant differentiation between lesions, PL, and NAWM in MS patients with relatively short disease duration on a group-level. The observed heterogeneity of qT1, qT2, qT2 * , PD, and QSM lesion values might reflect the biomarkers’ sensitivity to different lesion types, stages or degrees of de-/remyelination. Concerning diffuse WM pathology, the myelin-sensitive measures (MWF, MTsat, and ihMTR) appear to be the most promising MR biomarkers to separate NAWM from HCWM. Of note, concerning NAWM and NAGM, the presented results only allow for conclusions on a group-level. None of the investigated parameters demonstrated sufficiently large effects to allow assessment of NAWM in individual MS patients, due to the clearly overlapping value distribution of the 13 MS patients and 14 HC.

### Characterization of focal MS pathology (lesion and PL)

In general, a robust detection of lesions and PL is paramount to the assessment of MS using conventional, qualitative MRI [1]. Further characterization of lesion and PL with quantitative MR biomarkers might help to reveal interindividual differences between lesion types [3, 4].

In our study, on a group-level, all nine biomarkers showed significantly different values between lesions and NAWM, whereas only MTsat, ihMTR, qT2, PD, QSM, and T1w/T2w values were significantly different between lesions and NAGM. Detection of lesions within (cortical) GM is a well-known challenge, at least using 3 T scanners [32]. In each individual patient, particularly MTsat, PD, qT2, and T1w/T2w allowed for a robust delineation of MS lesions, showing a clear difference between lesion and PL values, thus potentially allowing for the definition of lesion cut-off values.

On a lesion-level, we looked more closely at the homogeneity of lesion biomarker values compared to surrounding tissue. Particularly qT1, qT2, qT2 * , PD, and QSM showed relatively higher variance of lesion values compared to surrounding tissue, whereas the variance of MWF, MTsat, ihMTR, and T1w/T2w within lesions was comparable to the signal variance of surrounding tissue [33]. Of note, in the present study, it is not really possible to disentangle (a) technical and (b) pathophysiological effects. One could argue that biomarkers showing relatively heterogeneous values both in lesions as well as in surrounding tissue might rather be influenced by technical effects, e.g., thermal noise. In addition, the assessed MR biomarkers might also be influenced to a varying extent by magnetic field inhomogeneities or might depend on the applied postprocessing routines, which affects the biomarker values globally, independent from the investigated tissue. In contrast, biomarkers showing heterogenous lesion values but homogeneous values in the surrounding tissue, likely reflect pathophysiological differences between lesions. The presence of a variety of pathophysiological alterations within MS lesions is key to the determination of different lesion types [3,34]. For example, QSM, which is sensitive to iron and myelin [5,19], is currently seen as a potential marker for identifying chronically active lesions [35–38], while qT1 potentially allows for a differentiation between lesions with destructive demyelination (long qT1) [39] and lesions with remyelination (short qT1) [40].

Regarding PL, all nine biomarkers revealed significantly different values compared to lesion and NAWM tissue on a group-level. Thereby, PL values were always between lesion and NAWM values, except for ihMTR (PL highest value) and QSM (PL lowest value). On a lesion-level, this effect is no longer detectable for ihMTR and becomes relatively small for QSM. Potential causes for lowest QSM values in the PL might be again due to a combination of (a) technical and (b) pathophysiological effects. a) In regions with high susceptibility, phase aliasing can be severe and phase fitting might not be successful with MEDI postprocessing. Additionally, in the surrounding of QSM lesions with high susceptibility, severe loss of signal (blooming artifacts) might be present, leading to an underestimation of PL QSM values [41]. b) QSM might also allow detecting molecular alterations in PL with relatively decreased susceptibility, such as remaining myelin around demyelinated lesions. Furthermore, iron loss has been shown to be pronounced towards the MS lesion edges [42], which could have caused the lower QSM values in PL tissue.

### Characterization of diffuse MS pathology within NAWM

Considering that the association between clinical findings and lesion volume is currently insufficient, the clinical relevance of detecting NAWM damage is becoming increasingly important [7,9]. NAWM damage precedes lesion formation [43] and might be of interest to differentiate between MS subtypes and to predict response to treatment.

In our study, the myelin-sensitive measures (MWF, MTsat, and ihMTR) were the most promising MR biomarkers to characterize NAWM compared to HCWM on a group-level, with the largest absolute differences between median values of MS patients and HC, albeit not statistically significant after correction for multiple testing. In contrast to other work, we analyzed whole-brain NAWM and HCWM VOIs instead of comparing scattered, manually defined regions of interest. Myelin-sensitive measures generally reveal reduced values in NAWM compared to HCWM, indicating myelin damage [22,23,25,44–46]. The rather new MT-based imaging technique ihMTR has been developed to further increase sensitivity and specificity to myelin changes compared to MTsat [15,44,47]. In our study of MS patients with relatively short disease duration and therefore presumably only mild changes in NAWM, ihMTR remains the only biomarker to detect significant differences between NAWM and HCWM. Despite the described trend, after correction for multiple testing, none of the assessed biomarkers could differentiate between NAWM and HCWM. In particular, the presented slight differences between WM tissue in MS patients and HC were only detectable on a group-level. None of the investigated biomarkers allowed for a characterization of abnormal WM changes in individual MS patients due to the clearly overlapping value distribution of the 13 MS patients and 14 HC. Pathological changes in affected brain tissue of MS patients are known to be more prominent in severely affected patients with long disease duration [48, 49], whereas our study focused on data from MS patients with relativity short disease duration and low EDSS. This might also contribute to the comparably small differences between NAWM and HCWM in our work.

### Reproducibility

To be able to relate MR biomarker changes to pathology or treatment effects rather than to inter-scan session variability, reproducibility assessment is crucial. Good multicenter reproducibility of MWF maps was shown for the applied GRASE sequence [50]. In previous work, we could also show high reproducibility of qT1, qT2 * , PD, and MTsat parameter maps, even when different imaging acceleration techniques were applied [51]. In their work, Gracien et al. showed good inter-scanner reproducibility of qT1, qT2, qT2 * , and PD, when identical sequences were used [52]. High reproducibility of QSM measurements with the applied MEDI reconstruction method has also been demonstrated before [53]. Further assessment of reproducibility will be the subject of future research with a tailored shorter imaging protocol to allow investigating a larger patient cohort.

### Limitations

Our study is not without limitations. First, we investigated a small number of MS patients and HC. However, our pilot study is meant to analyze and compare a large number of different biomarkers and focus on the MR biomarkers’ potential to assess effects in individual MS patients. The small cohort size allowed for an extensive scanning procedure in each participant. Assessing the potential of the presented MR biomarkers for monitoring disease progression and therapy in a broad patient cohort will be the subject of further research. Second, particularly for the differentiation between lesion, PL, NAWM/GM, and HCWM/GM, we analyzed mean values of each patient averaging all data within each VOI. Consequently, patients with more lesions were treated equally to patients with only a few lesions. Additionally, we treated grey and white matter as two single anatomical regions, not accounting for more specific regions within the tissue such as separately analyzing cortical and deep grey matter or fiber tracts within white matter. Third, an analysis of the effect of different lesion types on the MR biomarker signal was not a subject of the present study, although particularly qT1 and QSM are currently debated as promising biomarkers to distinguish lesion types. Fourth, the inherently different resolution of sequences limits matching image resolution across acquisitions. A related limitation, particularly for T1w/T2w, is that one echo of the (relatively low resolution) GRASE sequence was used as the T2w volume, rather than a high-resolution 3D T2w acquisition. Spatial smoothing and further matching of acquisition parameters might help to overcome these disadvantages in the future. Fifth, in our study we analyzed all nine biomarkers separately and did not apply a multivariate approach. Given that each MR biomarker used in the present study has different biophysical origins and interpretations, there might be complementary information spread across metrics that may be more sensitive to individual effects when combined [54, 55]. Therefore, a multivariate approach will be part of further research.

## Conclusion

With our prospective multi-parametric, quantitative MRI study, we intended to provide decision support for the implementation of quantitative MR biomarkers in the clinical routine assessment of MS patients. In the small study cohort of our clinical feasibility study, all biomarkers are suitable for MS lesions characterization in MS patients with relatively short disease duration. Thereby, the observed heterogeneity of qT1, qT2, qT2 * , PD, and QSM lesion values might reflect biological effects. A separation of NAWM and HCWM and, thus, characterization of diffuse pathological alterations in MS brain tissue could be most promising with the myelin-sensitive measures MWF, MTsat, and ihMTR. However, none of the investigated parameters appeared suitable for individual clinical diagnosis of pathological alterations in normal-appearing brain tissue, at least in our small cohort of MS patients with a short disease duration. The MR biomarkers’ potential for monitoring disease course and therapy effectiveness particularly in the course of a larger study has to be evaluated in the future.

## Supporting information

S1 Table Scan parameters of all acquired MR sequences. (PDF)

S1 Appendix Parameter map calculations. (PDF)

S2 Appendix Volume of interest definition. (PDF)

S1 Fig Lesion-level analysis of MR biomarkers. Lesion-level analysis showing the biomarker signal behavior from individual lesion to surrounding tissue plotted as individual lines with the y-axes scaled to the mean ±  twice the standard deviation of each MR biomarker. The red line indicates the mean signal across all lesions.
(PDF)

S2 Fig Graphical abstract of the performed study. (PDF)

## References

[1] Wattjes MP, Ciccarelli O, Reich DS, Banwell B, de Stefano N, Enzinger C, et al. 2021 MAGNIMS-CMSC-NAIMS consensus recommendations on the use of MRI in patients with multiple sclerosis. Lancet Neurol. 2021;20(8):653–70.doi: 10.1016/S1474-4422(21)00095-8 34139157

[2] Filippi M, Preziosa P, Banwell BL, Barkhof F, Ciccarelli O, De Stefano N, et al. Assessment of lesions on magnetic resonance imaging in multiple sclerosis: practical guidelines. Brain. 2019;142(7):1858–75.doi: 10.1093/brain/awz144 31209474 PMC6598631

[3] Kuhlmann T, Ludwin S, Prat A, Antel J, Brück W, Lassmann H. An updated histological classification system for multiple sclerosis lesions. Acta Neuropathol. 2017;133(1):13–24.doi: 10.1007/s00401-016-1653-y 27988845

[4] Frischer J, Weigand S, Guo Y, Kale N, Parisi J, Pirko I. Clinical and pathological insights into the dynamic nature of the white matter multiple sclerosis plaque. Annals of Neurology. 2015;78(5):710–21. 26239536 10.1002/ana.24497 PMC4623970

[5] Granziera C, Wuerfel J, Barkhof F, Calabrese M, De Stefano N, Enzinger C, et al. Quantitative magnetic resonance imaging towards clinical application in multiple sclerosis. Brain. 2021;144(5):1296–311.doi: 10.1093/brain/awab029 33970206 PMC8219362

[6] Tranfa M, Pontillo G, Petracca M, Brunetti A, Tedeschi E, Palma G, et al. Quantitative MRI in Multiple Sclerosis: From Theory to Application. AJNR Am J Neuroradiol. 2022;43(12):1688–95.doi: 10.3174/ajnr.A7536 35680161

[7] Allen IV, McQuaid S, Mirakhur M, Nevin G. Pathological abnormalities in the normal-appearing white matter in multiple sclerosis. Neurol Sci. 2001;22(2):141–4.doi: 10.1007/s100720170012 11603615

[8] Hametner S, Wimmer I, Haider L, Pfeifenbring S, Brück W, Lassmann H. Iron and neurodegeneration in the multiple sclerosis brain. Ann Neurol. 2013;74(6):848–61.doi: 10.1002/ana.23974 23868451 PMC4223935

[9] Kutzelnigg A, Lucchinetti CF, Stadelmann C, Brück W, Rauschka H, Bergmann M, et al. Cortical demyelination and diffuse white matter injury in multiple sclerosis. Brain. 2005;128(Pt 11):2705–12.doi: 10.1093/brain/awh641 16230320

[10] Dziedzic T, Metz I, Dallenga T, König FB, Müller S, Stadelmann C, et al. Wallerian degeneration: a major component of early axonal pathology in multiple sclerosis. Brain Pathol. 2010;20(5):976–85.doi: 10.1111/j.1750-3639.2010.00401.x 20477831 PMC8094657

[11] Lieury A, Chanal M, Androdias G, Reynolds R, Cavagna S, Giraudon P, et al. Tissue remodeling in periplaque regions of multiple sclerosis spinal cord lesions. Glia. 2014;62(10):1645–58.doi: 10.1002/glia.22705 24910450

[12] Pierpaoli C. Quantitative brain MRI. Top Magn Reson Imaging. 2010;21(2):63.doi: 10.1097/RMR.0b013e31821e56f8 21613871 PMC4819319

[13] MacKay A, Laule C, Vavasour I, Bjarnason T, Kolind S, Mädler B. Insights into brain microstructure from the T2 distribution. Magn Reson Imaging. 2006;24(4):515–25.doi: 10.1016/j.mri.2005.12.037 16677958

[14] Wolff SD, Balaban RS. Magnetization transfer contrast (MTC) and tissue water proton relaxation in vivo. Magn Reson Med. 1989;10(1):135–44.doi: 10.1002/mrm.1910100113 2547135

[15] Duhamel G, Prevost VH, Cayre M, Hertanu A, Mchinda S, Carvalho VN, et al. Validating the sensitivity of inhomogeneous magnetization transfer (ihMT) MRI to myelin with fluorescence microscopy. Neuroimage. 2019;199 289–303.doi: 10.1016/j.neuroimage.2019.05.061 31141736

[16] Schmierer K, Scaravilli F, Altmann DR, Barker GJ, Miller DH. Magnetization transfer ratio and myelin in postmortem multiple sclerosis brain. Ann Neurol. 2004;56(3):407–15.doi: 10.1002/ana.20202 15349868

[17] Schmierer K, Wheeler-Kingshott CAM, Tozer DJ, Boulby PA, Parkes HG, Yousry TA, et al. Quantitative magnetic resonance of postmortem multiple sclerosis brain before and after fixation. Magn Reson Med. 2008;59(2):268–77.doi: 10.1002/mrm.21487 18228601 PMC2241759

[18] Mezer A, Yeatman JD, Stikov N, Kay KN, Cho N-J, Dougherty RF, et al. Quantifying the local tissue volume and composition in individual brains with magnetic resonance imaging. Nat Med. 2013;19(12):1667–72.doi: 10.1038/nm.3390 24185694 PMC3855886

[19] Hametner S, Endmayr V, Deistung A, Palmrich P, Prihoda M, Haimburger E, et al. The influence of brain iron and myelin on magnetic susceptibility and effective transverse relaxation - A biochemical and histological validation study. Neuroimage. 2018;179 117–33.doi: 10.1016/j.neuroimage.2018.06.007 29890327

[20] Granziera C, Wuerfel J, Barkhof F, Calabrese M, De Stefano N, Enzinger C, et al. Quantitative magnetic resonance imaging towards clinical application in multiple sclerosis. Brain. 2021;144(5):1296–311.doi: 10.1093/brain/awab029 33970206 PMC8219362

[21] Mühlau M. T1/T2-weighted ratio is a surrogate marker of demyelination in multiple sclerosis: No. Mult Scler. 2022;28(3):355–6.doi: 10.1177/13524585211063622 35067108 PMC8894978

[22] Saccenti L, Hagiwara A, Andica C, Yokoyama K, Fujita S, Kato S, et al. Myelin Measurement Using Quantitative Magnetic Resonance Imaging: A Correlation Study Comparing Various Imaging Techniques in Patients with Multiple Sclerosis. Cells. 2020;9(2):393.doi: 10.3390/cells9020393 32046340 PMC7072333

[23] Lommers E, Simon J, Reuter G, Delrue G, Dive D, Degueldre C. Multiparameter MRI quantification of microstructural tissue alterations in multiple sclerosis. NeuroImage: Clinical. 2019;23:101879. 31176293 10.1016/j.nicl.2019.101879 PMC6555891

[24] Hagiwara A, Hori M, Yokoyama K, Takemura MY, Andica C, Kumamaru KK, et al. Utility of a multiparametric quantitative MRI model that assesses myelin and edema for evaluating plaques, periplaque white matter, and normal-appearing white matter in patients with multiple sclerosis: a feasibility study. Am J Neuroradiol. 2017;38(2):237–42.doi: 10.3174/ajnr.A4977 27789453 PMC7963826

[25] O’Muircheartaigh J, Vavasour I, Ljungberg E, Li DKB, Rauscher A, Levesque V, et al. Quantitative neuroimaging measures of myelin in the healthy brain and in multiple sclerosis. Hum Brain Mapp. 2019;40(7):2104–16.doi: 10.1002/hbm.24510 30648315 PMC6590140

[26] Rahmanzadeh R, Weigel M, Lu P-J, Melie-Garcia L, Nguyen TD, Cagol A, et al. A comparative assessment of myelin-sensitive measures in multiple sclerosis patients and healthy subjects. Neuroimage Clin. 2022;36 103177.doi: 10.1016/j.nicl.2022.103177 36067611 PMC9468574

[27] Prasloski T, Rauscher A, MacKay AL, Hodgson M, Vavasour IM, Laule C, et al. Rapid whole cerebrum myelin water imaging using a 3D GRASE sequence. Neuroimage. 2012;63(1):533–9.doi: 10.1016/j.neuroimage.2012.06.064 22776448

[28] Girard OM, Prevost VH, Varma G, Cozzone PJ, Alsop DC, Duhamel G. Magnetization transfer from inhomogeneously broadened lines (ihMT): experimental optimization of saturation parameters for human brain imaging at 1.5 Tesla. Magn Reson Med. 2015;73(6):2111–21.doi: 10.1002/mrm.25330 24962257

[29] Tabelow K, Balteau E, Ashburner J, Callaghan MF, Draganski B, Helms G, et al. hMRI - a toolbox for quantitative MRI in neuroscience and clinical research. Neuroimage. 2019;194:191–210.doi: 10.1016/j.neuroimage.2019.01.029 30677501 PMC6547054

[30] Liu J, Liu T, de Rochefort L, Ledoux J, Khalidov I, Chen W, et al. Morphology enabled dipole inversion for quantitative susceptibility mapping using structural consistency between the magnitude image and the susceptibility map. Neuroimage. 2012;59(3):2560–8.doi: 10.1016/j.neuroimage.2011.08.082 21925276 PMC3254812

[31] Li H, Jiang G, Zhang J, Wang R, Wang Z, Zheng W-S, et al. Fully convolutional network ensembles for white matter hyperintensities segmentation in MR images. Neuroimage. 2018;183:650–65.doi: 10.1016/j.neuroimage.2018.07.005 30125711

[32] Patel KR, Luo J, Alvarez E, Piccio L, Schmidt RE, Yablonskiy DA, et al. Detection of cortical lesions in multiple sclerosis: A new imaging approach. Mult Scler J Exp Transl Clin. 2015;1:2055217315606465.doi: 10.1177/2055217315606465 28607704 PMC5433400

[33] Johnson P, Vavasour IM, Stojkova BJ, Abel S, Lee LE, Laule C, et al. Myelin heterogeneity for assessing normal appearing white matter myelin damage in multiple sclerosis. J Neuroimaging. 2023;33(2):227–34.doi: 10.1111/jon.13069 36443960

[34] Lucchinetti C, Brück W, Parisi J, Scheithauer B, Rodriguez M, Lassmann H. Heterogeneity of multiple sclerosis lesions: implications for the pathogenesis of demyelination. Ann Neurol. 2000;47(6):707–17.doi: 10.1002/1531-8249(200006)47:6<707::aid-ana3>3.0.co;2-q 10852536

[35] Wisnieff C, Ramanan S, Olesik J, Gauthier S, Wang Y, Pitt D. Quantitative susceptibility mapping (QSM) of white matter multiple sclerosis lesions: interpreting positive susceptibility and the presence of iron. Magn Reson Med. 2015;74(2):564–70.doi: 10.1002/mrm.25420 25137340 PMC4333139

[36] Deh K, Ponath GD, Molvi Z, Parel G-CT, Gillen KM, Zhang S, et al. Magnetic susceptibility increases as diamagnetic molecules breakdown: Myelin digestion during multiple sclerosis lesion formation contributes to increase on QSM. J Magn Reson Imaging. 2018;48(5):1281–7.doi: 10.1002/jmri.25997 29517817 PMC6129234

[37] Zhang Y, Gauthier SA, Gupta A, Tu L, Comunale J, Chiang GC-Y, et al. Magnetic susceptibility from quantitative susceptibility mapping can differentiate new enhancing from nonenhancing multiple sclerosis lesions without gadolinium injection. Am J Neuroradiol. 2016;37(10):1794–9.doi: 10.3174/ajnr.A4856 27365331 PMC5201451

[38] Kaunzner UW, Kang Y, Zhang S, Morris E, Yao Y, Pandya S, et al. Quantitative susceptibility mapping identifies inflammation in a subset of chronic multiple sclerosis lesions. Brain. 2019;142(1):133–45.doi: 10.1093/brain/awy296 30561514 PMC6308309

[39] Brex PA, Parker GJ, Leary SM, Molyneux PD, Barker GJ, Davie CA, et al. Lesion heterogeneity in multiple sclerosis: a study of the relations between appearances on T1 weighted images, T1 relaxation times, and metabolite concentrations. J Neurol Neurosurg Psychiatry. 2000;68(5):627–32.doi: 10.1136/jnnp.68.5.627 10766895 PMC1736901

[40] Kolb H, Absinta M, Beck ES, Ha S-K, Song Y, Norato G, et al. 7T MRI Differentiates Remyelinated from Demyelinated Multiple Sclerosis Lesions. Ann Neurol. 2021;90(4):612–26.doi: 10.1002/ana.26194 34390015 PMC9291186

[41] Gharabaghi S, Liu S, Wang Y, Chen Y, Buch S, Jokar M, et al. Multi-echo quantitative susceptibility mapping for strategically acquired gradient echo (STAGE) imaging. Front Neurosci. 2020;14:581474.doi: 10.3389/fnins.2020.581474 33192267 PMC7645168

[42] Popescu BF, Frischer JM, Webb SM, Tham M, Adiele RC, Robinson CA, et al. Pathogenic implications of distinct patterns of iron and zinc in chronic MS lesions. Acta Neuropathol. 2017;134(1):45–64.doi: 10.1007/s00401-017-1696-8 28332093 PMC5486634

[43] Filippi M, Rocca MA, Martino G, Horsfield MA, Comi G. Magnetization transfer changes in the normal appearing white matter precede the appearance of enhancing lesions in patients with multiple sclerosis. Ann Neurol. 1998;43(6):809–14. doi: 10.1002/ana.410430616 9629851

[44] Van Obberghen E, Mchinda S, le Troter A, Prevost VH, Viout P, Guye M, et al. Evaluation of the sensitivity of inhomogeneous magnetization transfer (ihMT) MRI for multiple sclerosis. Am J Neuroradiol. 2018;39(4):634–41.doi: 10.3174/ajnr.A5563 29472299 PMC7410781

[45] Faizy TD, Thaler C, Kumar D, Sedlacik J, Broocks G, Grosser M, et al. Heterogeneity of multiple sclerosis lesions in multislice myelin water imaging. PLoS One. 2016;11(3):e0151496.doi: 10.1371/journal.pone.0151496 26990645 PMC4798764

[46] Oh J, Han ET, Lee MC, Nelson SJ, Pelletier D. Multislice brain myelin water fractions at 3T in multiple sclerosis. J Neuroimag. 2007;17(2):156–63. 10.1111/j.1552-6569.2007.00098.x 17441837

[47] Ercan E, Varma G, Mädler B, Dimitrov I, Pinho M, Xi Y, et al. Microstructural correlates of 3D steady-state inhomogeneous magnetization transfer (ihMT) in the human brain white matter assessed by myelin water imaging and diffusion tensor imaging. Magnetic Resonance in Medicine. 2018;80(6):2402–14. 29707813 10.1002/mrm.27211

[48] Vrenken H, Geurts JJG, Knol DL, Polman CH, Castelijns JA, Pouwels PJW, et al. Normal-appearing white matter changes vary with distance to lesions in multiple sclerosis. Am J Neuroradiol. 2006;27(9):2005–11. 17032884 PMC7977884

[49] Vrenken H, Geurts JJG, Knol DL, van Dijk LN, Dattola V, Jasperse B, et al. Whole-brain T1 mapping in multiple sclerosis: global changes of normal-appearing gray and white matter. Radiology. 2006;240(3):811–20.doi: 10.1148/radiol.2403050569 16868279

[50] Lee LE, Ljungberg E, Shin D, Figley CR, Vavasour IM, Rauscher A, et al. Inter-vendor reproducibility of myelin water imaging using a 3D gradient and spin echo sequence. Front Neurosci. 2018;12:854. 30519158 10.3389/fnins.2018.00854 PMC6258882

[51] Berg RC, Leutritz T, Weiskopf N, Preibisch C. Multi-parameter quantitative mapping of R1, R2*, PD, and MTsat is reproducible when accelerated with Compressed SENSE. Neuroimage. 2022;253:119092.doi: 10.1016/j.neuroimage.2022.119092 35288281

[52] Gracien R-M, Maiworm M, Brüche N, Shrestha M, Nöth U, Hattingen E, et al. How stable is quantitative MRI? - assessment of intra- and inter-scanner-model reproducibility using identical acquisition sequences and data analysis programs. Neuroimage. 2020;207 116364.doi: 10.1016/j.neuroimage.2019.116364 31740340

[53] Deh K, Kawaji K, Bulk M, Van Der Weerd L, Lind E, Spincemaille P, et al. Multicenter reproducibility of quantitative susceptibility mapping in a gadolinium phantom using MEDI+0 automatic zero referencing. Magn Reson Med. 2019;81(2):1229–36.doi: 10.1002/mrm.27410 30284727 PMC6289704

[54] Tardif CL, Gauthier CJ, Steele CJ, Bazin P-L, Schäfer A, Schaefer A, et al. Advanced MRI techniques to improve our understanding of experience-induced neuroplasticity. Neuroimage. 2016;131 55–72.doi: 10.1016/j.neuroimage.2015.08.047 26318050

[55] Lambert C, Chowdhury R, Fitzgerald THB, Fleming SM, Lutti A, Hutton C, et al. Characterizing aging in the human brainstem using quantitative multimodal MRI analysis. Front Hum Neurosci. 2013;7 462.doi: 10.3389/fnhum.2013.00462 23970860 PMC3747448

