# Supplementary material for: Sensitivity of multi-parametric quantitative magnetic resonance imaging for multiple sclerosis pathology
Source: PLoS One. 2025 Apr 16;20(4):e0318415. doi: 10.1371/journal.pone.0318415 (PMC12002544; doi:10.1371/journal.pone.0318415)
Supplement: S1 Table — (PDF) [file pone.0318415.s001.pdf]

Supplementary Material

Table S1: Scan parameters of all acquired MR sequences

	**FLAIR**	**MPRAGE**	**MWI**	**ihMT**	**B1 map**	**T1w**	**PDw**	**MTw**	**SWI**
Image type	3D TSE	FFE (TFE)	(GRASE)	FFE (EPI)	3D FFE	3D FFE	3D FFE	3D FFE	3D FFE
Resolution [mm³]	$(1.2)^3$	$(1.0)^3$	1x2x5	$(2.2)^3$	.5x3.5 x5	$(1.0)^3$	$(1.0)^3$	$(1.0)^3$	x0.7x1.4
FoV [mm³]	249 x168	240 x161	192 x100	211x161	240 x175	240 x176	240 x176	240 x176	193x140
# slices	140	161	40	73	70	176	176	176	100
# echoes	1	1	48	3	1	6	6	6	6
. / ΔTE [ms]	276	3.7	8 / 8	3.5/5.7	2.3	2.4 / 2.4	2.4 / 2.4	2.4 / 2.4	6/6
TR [ms]	4800	8.1	1120	108	30 / 150	18	18	35	
lip angle [°]	90	8	90	15	60	25	4	6	12
ter-fat shift [pix]	0.386	2.268	2.263	1.984	0.870	0.901	0.901	0.901	ninimum
/TFE factor	170	159	48	-	-	-	-	-	-
EPI factor	-	-	3	5	-	-	-	-	-
MB factor	-	-	-	-	-	-	-	-	-
Halfscan	no	no	no	no	no	: 0.6, Z:1	.625, Z:1	: 0.6, Z:1	no
SENSE/CS	CS: 5	CS: 3	SEN: 2	SEN: 2.4	CS: 6	CS: 6	CS: 6	CS: 6	CS:4

fold-over	AP	AP	RL	RL	AP	AP	AP	AP	RL
fat shift dir.	F	F	L	L	F	F	F	F	P
an duration [min:s]	04:24	03:42	07:50	05:45	03:04	01:34	01:39	03:04	04:59

Table S1: Scan parameters of all acquired MR sequences for both lesion segmentation and parameter quantification. AP, anterior-posterior; CS, Compressed SENSE; dir, direction; EPI, echo-planar imaging; FLAIR, fluid-attenuated inversion recovery; F, foot; FFE, fast field echo; FoV, field of view; GRASE, gradient and spin echo; ihMT, inhomogeneous magnetization transfer; L, left; MB, multi band; MPRAGE, magnetization prepared rapid gradient echo; MT, magnetization transfer; MWI, myelin water imaging; P, posterior; PD, proton density; RL, right-left; SE, spin echo; SEN, SENSE; SWI, susceptibility-weighted imaging; TE, echo time; TFE, turbo field echo; TR, repetition time; TSE, turbo spin echo. The fold-over direction is the primary phase-encoding direction.
