# Supplementary material for: Sensitivity of multi-parametric quantitative magnetic resonance imaging for multiple sclerosis pathology
Source: PLoS One. 2025 Apr 16;20(4):e0318415. doi: 10.1371/journal.pone.0318415 (PMC12002544; doi:10.1371/journal.pone.0318415)
Supplement: S1 Appendix — (PDF) [file pone.0318415.s002.pdf]

Supplementary Material

Appendix S2: Parameter map calculations

The myelin water fraction (MWF) was determined by multi-exponential fitting procedures from the 3D gradient and spine echo (GRASE) data using a Sparsity Promoting Iterative Joint Non-negative least squares (SPIJN) algorithm for MWF determination published by Nagtegaal et al. (Nagtegaal et al., 2020; DOI: 10.1016/j.neuroimage.2020.117014).

Inhomogenous MT ratios (ihMTR) were calculated based on the four different 3D gradient-echo-based magnetization transfer (MT)-weighted images with single (positive and negative) and dual MT saturation (MTsat) pulse offset frequencies according to Girard et al. (Girard et al., 2015; DOI: 10.1002/mrm.25330).

Quantitative T1 (qT1), T2* (qT2*), proton density (PD), and MTsat parameter maps were generated from the 3D multi-echo gradient echo data using the hMRI toolbox (Tabelow et al., 2019; DOI: 10.1016/j.neuroimage.2019.01.029) included in the SPM framework (SPM12, version v7771; www.fil.ion.ucl.ac.uk/spm/software/spm12/) via the variable flip angle (VFA) approach (Baudrexel et al., 2018; DOI: 10.1002/mrm.26979; Preibisch and Deichmann, 2009; DOI: 10.1002/mrm.21969). Information from all three multi-echo contrasts were combined for estimation of qT2* (Weiskopf et al., 2014; DOI: 10.3389/fnins.2014.00278). Default configuration parameters were used except for a threshold of 10^8 for the PD map before bias field correction (which is scanner dependent) and a threshold of 15 percent units (p.u.) for the MTsat values (which depends on the utilized MT parameters) (Berg et al., 2022; DOI: 10.1016/j.neuroimage.2022.119750). Additionally, B1 errors were corrected via a model-based approach (Rowley et al., 2021; DOI: 10.1002/mrm.28831). All 0-values in the qT1 and qT2* parameter maps were considered unrealistic and thus set to “NaN”.

Quantitative T2 (qT2) parameter maps were generated via fitting of the 48-echo 3D GRASE 3D data that were also used for MWI.

Quantitative susceptibility mapping (QSM) data sets were generated via the MEDI processing (http://pre.weill.cornell.edu/mri/pages/qsm.html) as described in the work by Berg et al. (Berg et al., 2021; DOI: 10.1016/j.neuroimage.2021.118399).

Calculation of the T1w/T2w ratio was performed by taking the magnetization prepared rapid gradient echo (MPRAGE) as T1-weighted (T1w) images and the 15th echo of the 3D GRASE data (TE = 120 ms) as T2-weighted (T2w) image. T1w was then divided by a scaled T2w image, where the scaling factor s is calculated from the average T1w and T2w values in whole-brain grey matter (GM):

$$s = avg(T1w_{GM})/avg(T2w_{GM})$$

$$T1w/T2w = T1w/(s*T2w)$$
