# Supplementary material for: Sensitivity of multi-parametric quantitative magnetic resonance imaging for multiple sclerosis pathology
Source: PLoS One. 2025 Apr 16;20(4):e0318415. doi: 10.1371/journal.pone.0318415 (PMC12002544; doi:10.1371/journal.pone.0318415)
Supplement: S2 Appendix — (PDF) [file pone.0318415.s003.pdf]

Supplementary Material

Appendix S3: Volume of interest definition

Lesions:

Lesions, defined as white matter hyperintensities on fluid-attenuated inversion recovery images, were automatically segmented using an in-house built patch-based U-net. Lesions with a volume of 15 voxels or less were excluded. Individual lesions were obtained using MATLAB's "bwconncomp" function, which was applied to find connected components within the lesion mask. The resulting individual lesions were eroded with a sphere-shaped structuring element of radius one voxel to reduce partial volume effects caused by the registration of data sets and by differences in spatial resolution. Furthermore, cerebrospinal fluid (CSF) segmentations dilated with a sphere-shaped structuring element of radius two voxels were subtracted from the lesion volume of interests (VOIs) to remove partial volume influences from CSF.

Lesion-related volumes of interest:

In order to define the perilesion (PL) region, first MATLAB's "imdilate" function was applied to the lesion mask with a sphere-shaped structuring element of radius two voxels. Next, the lesion voxels were excluded from the obtained PL mask. Finally, this mask was intersected with whole-brain white matter (WM) VOI to obtain the PL tissue within normal-appearing white matter (NAWM).

Shell 1-3 represent one-voxel-wide shells around the PL VOI. MATLAB's "imdilate" function was applied to the PL mask with a sphere-shaped structuring element of radius n voxel(s), where n assumes the values 1-3. Lesion mask, PL mask and (for n = 2 and n = 3) shell mask n-1 were excluded, and the resulting shell mask was intersected with the whole-brain WM segmentation.
