# Supplementary material for: Sensitivity of multi-parametric quantitative magnetic resonance imaging for multiple sclerosis pathology
Source: PLoS One. 2025 Apr 16;20(4):e0318415. doi: 10.1371/journal.pone.0318415 (PMC12002544; doi:10.1371/journal.pone.0318415)
Supplement: S1 Fig — The red line indicates the mean signal across all lesions. (PDF) [file pone.0318415.s004.pdf]

Supplementary Material

Fig S4: Lesion-level analysis of MR biomarkers

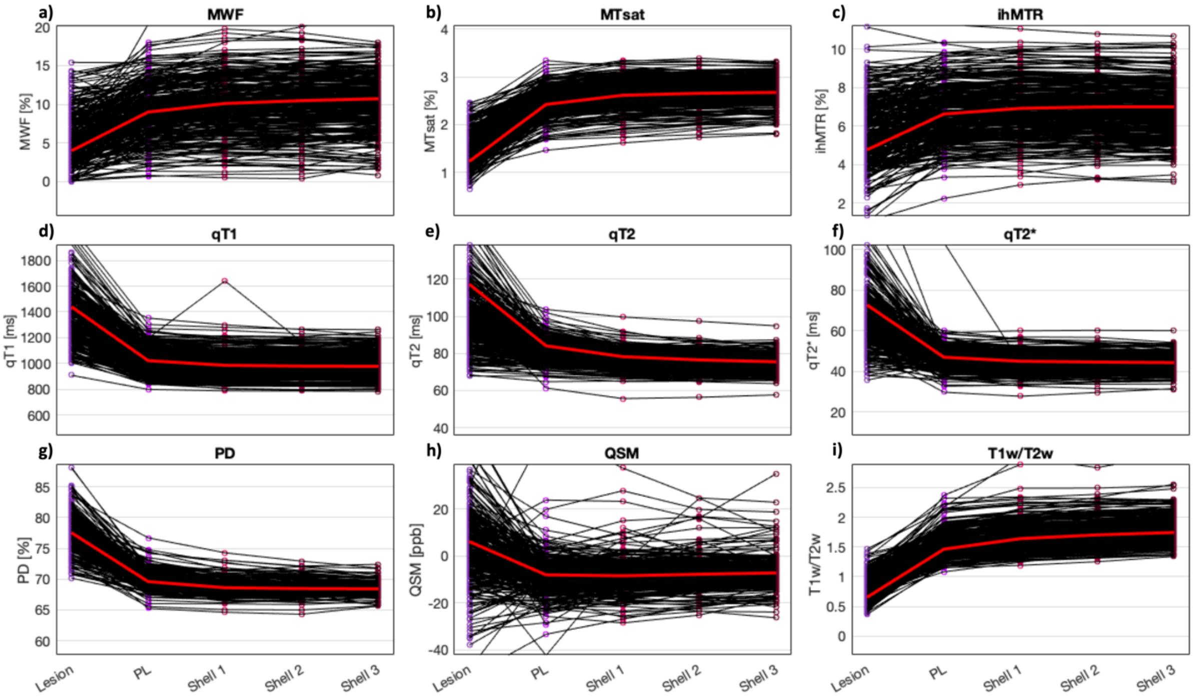

Fig S4. Lesion-level analysis showing the biomarker signal behavior from individual lesion to surrounding tissue plotted as individual lines with the y-axes scaled to the mean ± twice the standard deviation of each MR biomarker. The red line indicates the mean signal across all lesions. ihMTR, inhomogeneous MT ratio; MTsat, magnetization transfer saturation; MWF, myelin water fraction; NAWM, normal-appearing white matter; PL, perilesion; PD, proton density; QSM, quantitative susceptibility mapping; T1w/T2w, ratio between T1-weighted and T2-weighted images; VOI, volume of interest.
