# Supplementary figures and images for: Sensitivity of multi-parametric quantitative magnetic resonance imaging for multiple sclerosis pathology
Source: PLoS One. 2025 Apr 16;20(4):e0318415. doi: 10.1371/journal.pone.0318415 (PMC12002544; doi:10.1371/journal.pone.0318415)

## 9 MR biomarkers

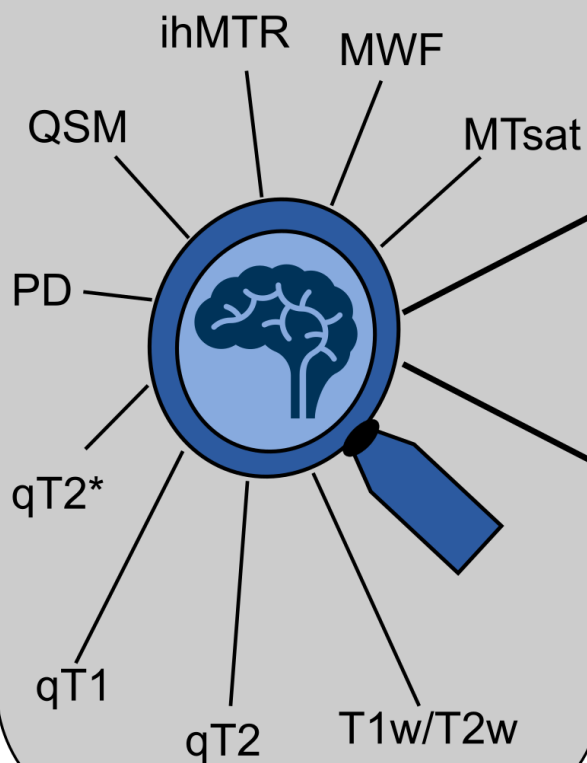

## Volumes of interest

14 Healthy

HCWM

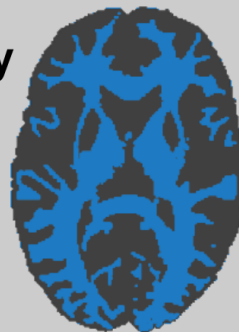

13 MS (early-stage)

NAWM

Lesion

Perilesion

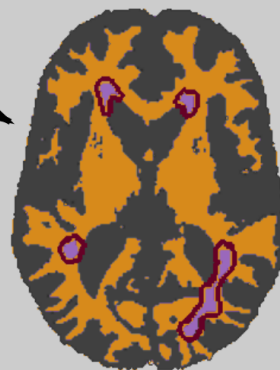

## Results

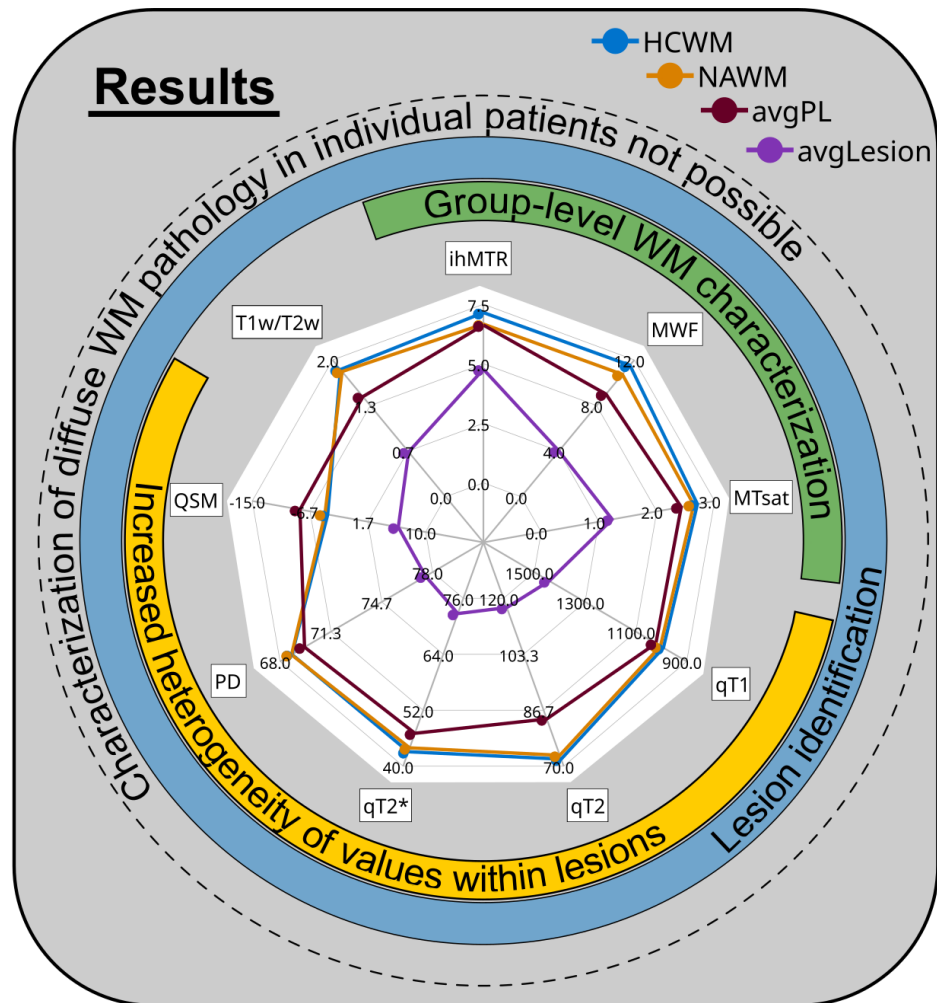

Supplement: S2 Fig — (PDF) [file pone.0318415.s005.pdf]
